# Beyond infection: Ommaya reservoir-induced vasogenic edema and reversible leukoencephalopathy

**DOI:** 10.1093/omcr/omaf195

**Published:** 2025-10-22

**Authors:** Mohamed Fadil, Imane Mohammadine, Rachida Saouab, Hassan En-Nouali, Jamal El Fenni, Zakariya Toufga

**Affiliations:** Radiology department, Mohamed V Military Hospital, Av. Abderrahim Bouabid, Rabat 10112, Morocco; Radiology department, Mohamed V Military Hospital, Av. Abderrahim Bouabid, Rabat 10112, Morocco; Radiology department, Mohamed V Military Hospital, Av. Abderrahim Bouabid, Rabat 10112, Morocco; Radiology department, Mohamed V Military Hospital, Av. Abderrahim Bouabid, Rabat 10112, Morocco; Radiology department, Mohamed V Military Hospital, Av. Abderrahim Bouabid, Rabat 10112, Morocco; Radiology department, Mohamed V Military Hospital, Av. Abderrahim Bouabid, Rabat 10112, Morocco

**Keywords:** Ommaya reservoir, MRI, leukoencephalopathy, Edema

## Abstract

Ommaya reservoirs, commonly used in the management of intrathecal chemotherapy, can sometimes lead to complications, including cerebral edema and porencephaly. We present the case of a 65-year-old woman with a suprasellar craniopharyngioma who developed these rare complications after the placement of an Ommaya reservoir. The initial CT and MRI findings revealed a hypodense area in the right frontal lobe along the Ommaya catheter, consistent with a vasogenic edema. Laboratory work-ups, including inflammatory markers, were normal, and the patient did not exhibit fever. This case highlights the need for clinicians to be aware of this potential complication when managing patients with Ommaya reservoirs.

## Introduction

Ommaya reservoirs are primarily used to deliver intrathecal chemotherapy for patients with primary or metastatic central nervous system tumors, but they are also frequently employed for cerebrospinal fluid drainage in conditions such as hydrocephalus. Though effective in treating malignant brain lesions, these reservoirs can cause various complications, including infection, catheter obstruction, and, more rarely, neurological issues such as cerebral edema and porencephaly [1, 2]. The pathophysiology of these complications remains poorly understood, but it is hypothesized that the mechanical pressure from the catheter may disrupt the normal brain architecture [3]. Here, we describe a rare case of cerebral edema associated with an Ommaya reservoir in a patient with a suprasellar craniopharyngioma.

## Case report

A 65-year-old woman with a history of suprasellar craniopharyngioma, previously treated with surgery and radiation therapy, presented with progressively worsening left-sided hemiparesis over the course of three weeks. She had a history of a prior Ommaya reservoir placement three years ago for the drainage of cerebrospinal fluid (CSF) due to hydrocephalus caused by her craniopharyngioma.

**Figure 1:**
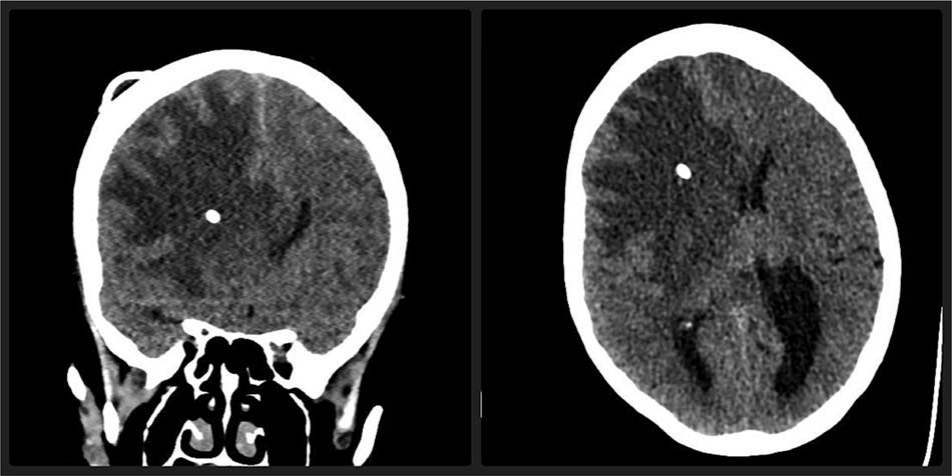
Axial and coronal non-contrast CT scan of the brain demonstrate a marked hypodense area in the right frontal lobe, predominantly involving the white matter, associated with significant mass effect and midline shift. Note the hyperdense catheter inside brain parenchyma.

**Figure 2:**
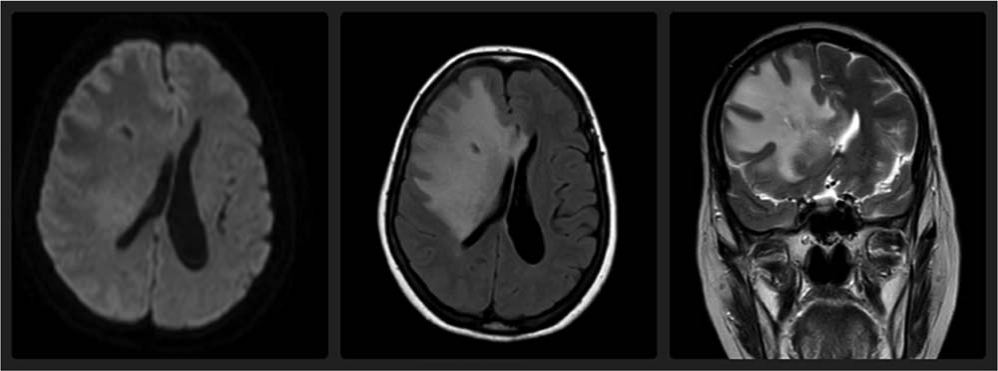
Axial FLAIR, axial DWI, and coronal T2-weighted MRI demonstrate a hyperintense signal in the right frontal lobe white matter on FLAIR and T2 sequences, without diffusion restriction, consistent with vasogenic edema and early leukoencephalopathy.

Upon admission, a non-contrast CT scan of the brain revealed a hypodense area in the right frontal lobe along the Ommaya catheter (Fig. 1). To further assess this finding, an MRI was performed, which demonstrated an increased T2 and FLAIR signal in the white matter of the right frontal lobe, suggesting vasogenic edema. No diffusion restriction was noted, which helped differentiate this from cytotoxic edema (Fig. 2) [4]. Laboratory work-ups, including inflammatory markers (C-reactive protein, erythrocyte sedimentation rate, and white blood cell count), were all within normal limits. Cerebrospinal fluid (CSF) analysis was also performed, including bacterial cultures and viral PCRs, all of which returned negative. Notably, the patient did not exhibit any signs of systemic infection, such as fever or leukocytosis, ruling out CNS (central nervous system) infection.

**Figure 3:**
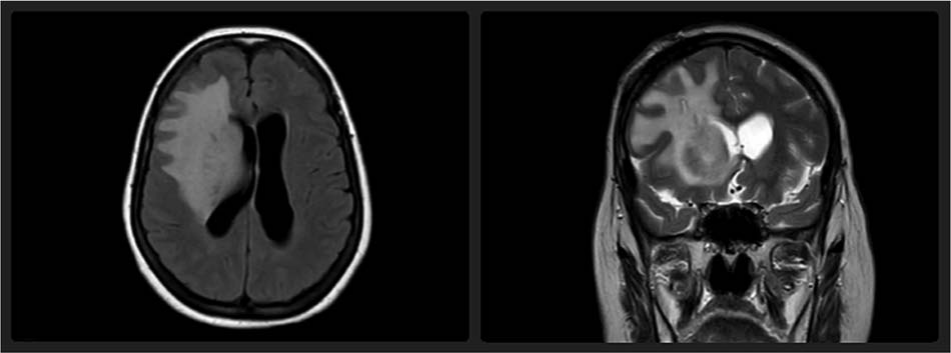
Axial FLAIR and coronal T2-weighted MRI performed three weeks after catheter removal demonstrate a notable reduction in the previously observed hyperintensity in the right frontal lobe, indicating partial resolution of the leukoencephalopathy.

Over the course of her hospitalization, the patient's neurological status fluctuated but did not deteriorate further. Following a multidisciplinary staff meeting, the neurosurgical team decided to remove the Ommaya catheter. The patient did not receive any medical treatment, including antibiotics. After catheter removal, she demonstrated progressive and partial neurological improvement, with partial recovery of motor function and attenuation of her left-sided hemiparesis. A follow-up MRI performed three weeks later revealed a reduction in the leukoencephalopathy, consistent with resolution of the edema (Fig. 3).

## Discussion

Ommaya reservoirs are commonly used in the management of intrathecal chemotherapy patients, most often in the situation of primary or metastatic central nervous system (CNS) tumors. While the devices are generally safe and effective for long-term cerebrospinal fluid (CSF) drainage and drug administration, they are not without complications. The most common adverse effects of Ommaya reservoirs are infection, catheter occlusion, and mechanical failure [1]. However, as in this instance, less frequent and less reported complications, such as cerebral edema and porencephaly, can also happen, presenting diagnostic and therapeutic challenges.

The exact mechanisms of cerebral edema secondary to Ommaya reservoirs are unknown. One theoretical hypothesis is mechanical disruption of the brain parenchyma by the catheter itself. The inserted foreign body within the CSF space is able to locally cause tissue damage, and it has the capability of resulting in inflammatory reaction leading to the culmination in developing vasogenic edema [2]. Vasogenic edema occurs because the disruption in the blood–brain barrier takes place when the interstitial space in the brain has plasma proteins and water leaked into it. This edema occurs frequently with focal neurological deficits, as in our patient, and typically presents in T2 and FLAIR MRI sequences, which are characterized by hyperintense signals in the affected brain regions [3].

The other theory is the chronic irritation of the cerebral tissue by the reservoir catheter or by the infusate in the reservoir. This inflammation may lead to a slow, low-grade inflammatory response that ultimately results in increased vascular permeability and fluid buildup in the brain tissue. This hypothesis is in agreement with the resolution of edema following catheter removal, as withdrawal of the mechanical trauma to the brain may allow for restoration of normal vascular function and edema reduction [4]. Given the patient’s history of prior radiation therapy, it is plausible that radiation-induced changes in vascular integrity and white matter architecture may have predisposed her to an exaggerated inflammatory or vasogenic response to the Ommaya catheter. Radiation can cause chronic microvascular damage and disrupt the blood–brain barrier, potentially increasing susceptibility to edema formation in the presence of mechanical irritation or altered cerebrospinal fluid dynamics.

Furthermore, some evidence suggests that the volume of CSF aspirated or infusion rate through the Ommaya reservoir could have a role to play in cerebral edema formation. Disruption of CSF dynamics, particularly in patients who have an underlying condition such as hydrocephalus, could perhaps predispose to the formation of these neurological complications. In our patient, the patient's underlying hydrocephalus secondary to her suprasellar craniopharyngioma potentially predisposed her to these changes, with the Ommaya catheter potentially changing CSF pressure dynamics in a way that favored edema development.

One of the most challenging aspects of diagnosing cerebral edema secondary to Ommaya reservoirs is differentiating it from other more common causes of neurological deterioration, such as infection or tumor growth. In the absence of fever and if inflammatory markers (e.g. C-reactive protein, erythrocyte sedimentation rate, and white blood cell count) were within normal limits, an infectious etiology was less likely. This is a critical consideration since infections, particularly meningitis or ventriculitis, are usually the first thing to consider when a patient with an Ommaya reservoir develops new neurological deficits. In our patient, the lack of fever, along with the normal laboratory studies, made it extremely likely that the cause of her neurological deficits was not infectious.

Imaging plays a critical role in the diagnosis of cerebral edema and other complications of Ommaya reservoir. In this case, the initial CT scan showed a hypodense area along the Ommaya catheter in the right frontal lobe. The CT hypodensity indicated edema but was not specific to differentiate between vasogenic and cytotoxic edema. MRI, particularly with T2-weighted and FLAIR sequences, provided more diagnostic specificity. The hyperintensity on the white matter of the right frontal lobe was consistent with vasogenic edema, and the absence of restriction of diffusion on DWI ruled out cytotoxic edema, which is commonly associated with ischemic injury [5]. The MRI also did not reveal any mass effect, ruling out tumor expansion or hemorrhage as the cause of the patient's symptoms.

The following MRI three weeks after Ommaya catheter removal showed a dramatic reduction in the leukoencephalopathy, again supporting the hypothesis that the mechanical and/or inflammatory damage inflicted by the catheter was the cause of the cerebral edema. The reversibility of the edema after catheter removal demonstrates the reversible nature of this complication and suggests that prompt intervention can prevent long-term neurological damage.

This case also highlights the importance of consideration of less common complications of Ommaya reservoirs in the diagnosis of neurological symptoms in patients with such devices. Although infection remains the most concerning complication, other etiologies such as non-infectious causes such as cerebral edema, porencephaly, and other inflammatory or mechanical complications should also be considered. Imaging and clinical examination urgently conducted are key in the detection of such complications at an early time and in distinguishing such etiologies from more common factors of neurological deterioration.

The absence of fever and a normal inflammatory marker in this case substantiates the need for clinicians to broaden their differential diagnosis when treating patients with Ommaya reservoirs. It also emphasizes the importance of not exclusively relying on lab results when determining a patient's neurological status. Imaging must always be performed to search for structural changes, especially if clinical presentation is unusual or if infection is not a prime consideration.

Treatment of cerebral edema that occurs with Ommaya reservoirs is primarily removal of the catheter, and this was done in this patient and led to a response in the patient's neurological status. Observation with serial imaging may be appropriate if no immediate neurological deterioration is seen in order to ensure that the edema does not persist without needing more aggressive treatment such as steroids or osmotic therapy. However, if the edema increases or irreversible neurological damage is present, more invasive surgeries will be necessitated [6].

The prognosis for patients with complications like cerebral edema due to Ommaya reservoirs is generally good if detected early and managed accordingly. As seen in this case, the patient's condition improved significantly after catheter removal, with subsequent imaging showing improvement of the leukoencephalopathy. This suggests that with early treatment, neurological complications of Ommaya-related issues can be reversed.

## Conclusion

In conclusion, while Ommaya reservoirs are invaluable tools in the management of CNS malignancies, clinicians must remain vigilant for potential complications, including cerebral edema and porencephaly, which may occur even in the absence of overt infection. This case demonstrates the importance of a high index of suspicion and the need for comprehensive imaging and laboratory work-up to accurately diagnose non-infectious complications. Early recognition and intervention are critical in preventing irreversible neurological damage and ensuring the best possible outcomes for patients undergoing intrathecal chemotherapy.

## Data Availability

Not applicable for this case report.
